# Supplementary material for: Breaking the nanoparticle’s dispersible limit via rotatable surface ligands
Source: Nat Commun. 2022 Jun 23;13:3581. doi: 10.1038/s41467-022-31275-7 (PMC9226028; doi:10.1038/s41467-022-31275-7)
Supplement: Supplementary file 1 — Supplementary Information [file 41467_2022_31275_MOESM1_ESM.pdf]

Breaking the nanoparticle's dispersible limit via rotatable surface ligands

Yue Liu[1], Na Peng[2,3], Yifeng Yao[4], Xuan Zhang[4], Xianqi Peng[2], Liyan Zhao[1], Jing Wang[5], Liang Peng[6], Zuankai Wang[6], Kenji Mochizuki[4]*, Min Yue[2,3,7]*, and Shikuan Yang[1,7,8,9]*

[1]Institute for Composites Science Innovation, School of Materials Science and Engineering, Zhejiang University, Hangzhou 310027, China

[2]Institute of Veterinary Sciences & Department of Veterinary Medicine, College of Animal Sciences, Zhejiang University, Hangzhou 310058, China

[3]Hainan Institute of Zhejiang University, Sanya 572025, China

[4]Department of Chemistry, Zhejiang University, Hangzhou 310028, China

[5]Department of Mechanical Engineering, University of Michigan, Ann Arbor, Michigan 48109, United States

[6]Department of Mechanical Engineering, City University of Hong Kong, Hong Kong 999077, China

[7]Department of Medical Oncology, The first affiliated Hospital, Zhejiang University School of Medicine, Hangzhou 310003, China

[8]State Key Laboratory of Fluid Power and Mechatronic Systems, Zhejiang University, Hangzhou 310027, China

[9]Baotou Research Institute of Rare Earths, Baotou 014030, China

*Corresponding author. Email: kenji_mochizuki@zju.edu.cn; myue@zju.edu.cn; shkyang@zju.edu.cn

Supplementary Figures

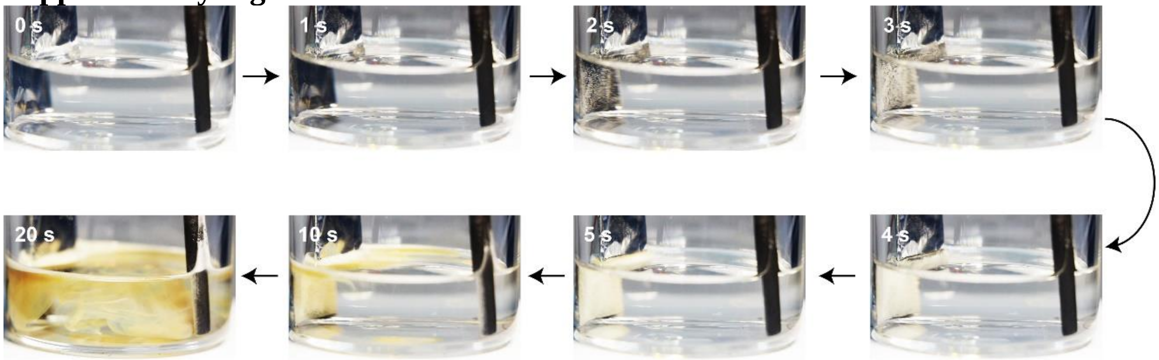

Supplementary Fig. 1. The formation process of smart Ag nanoparticles in the electrolyte solution at 30 V. The photos (captured from Supplementary Movie 1) of the electrolyte after electrodeposition for different times were shown.

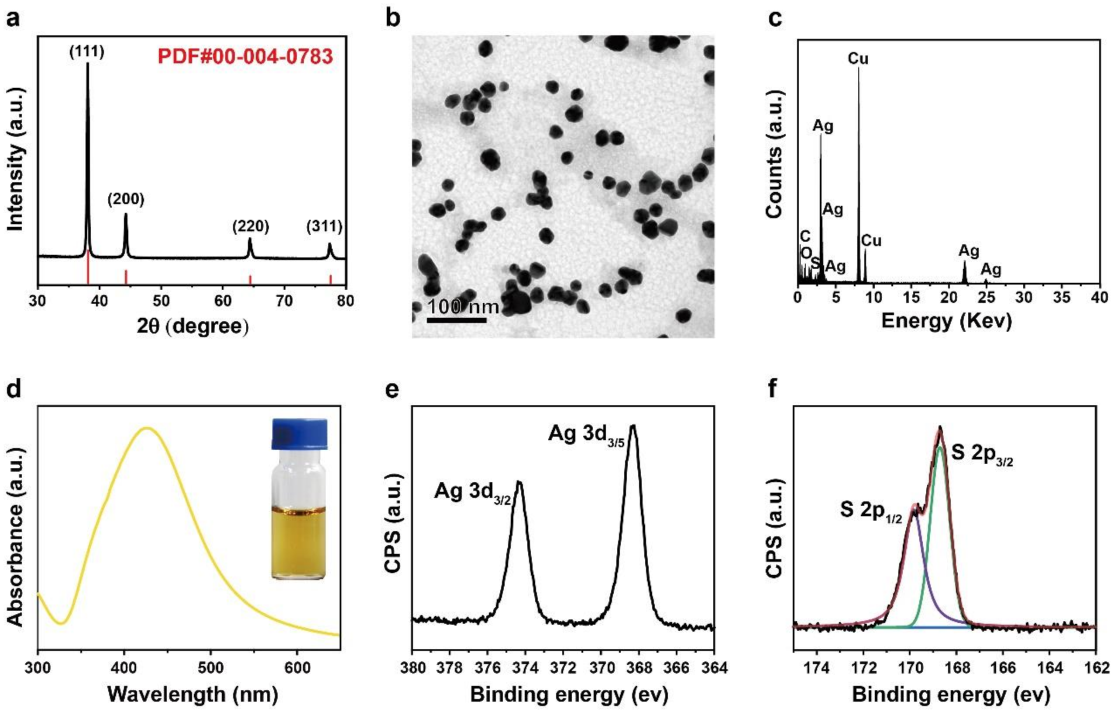

Supplementary Fig. 2. Characterization of the electrochemically prepared smart Ag nanoparticles. **a**, XRD pattern. **b**, TEM image used for energy dispersive spectrometer (EDS) analysis. **c**, EDS spectrum (Cu and C are from the carbon-coated copper grid used for TEM characterization). **d**, Absorption spectrum of the Ag nanoparticles dispersed in water. Inset: Photo of the colloidal solution. **e**, **f**, The binding energy of silver and sulfur in the Ag nanoparticles, respectively.

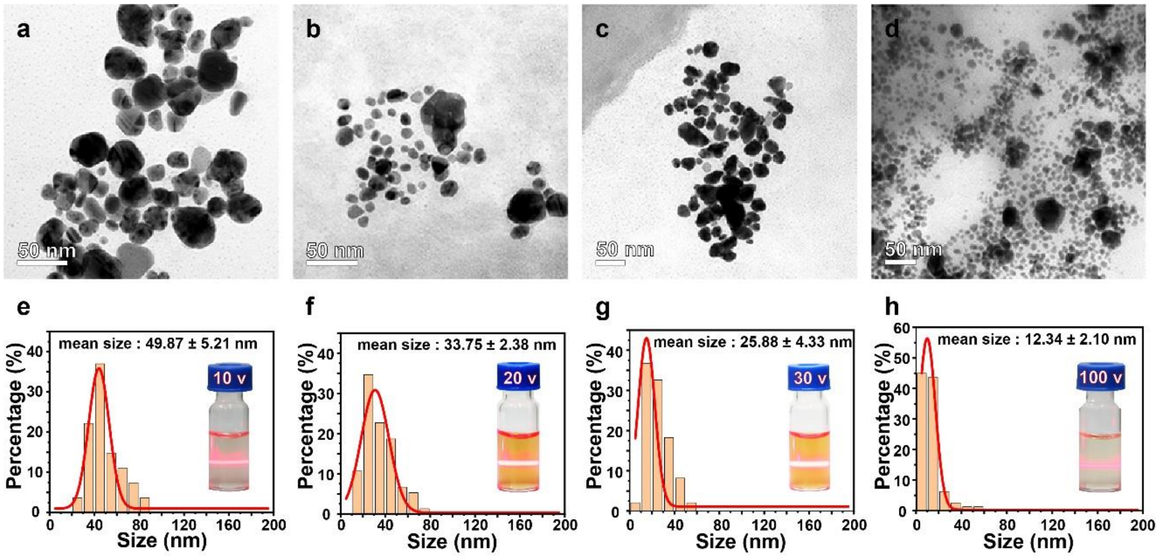

Supplementary Fig. 3. Morphology and size distribution of the smart Ag nanoparticles synthesized at different voltages. a to **d**, TEM images of Ag nanoparticles synthesized at 10 V, 20 V, 30 V, and 100 V, respectively. **e** to **h**, Size distribution of the Ag nanoparticles based on the measurement of > 100 nanoparticles synthesized at 10 V, 20 V, 30 V, and 100 V, respectively. Inset: Tyndall effect of the colloidal solutions.

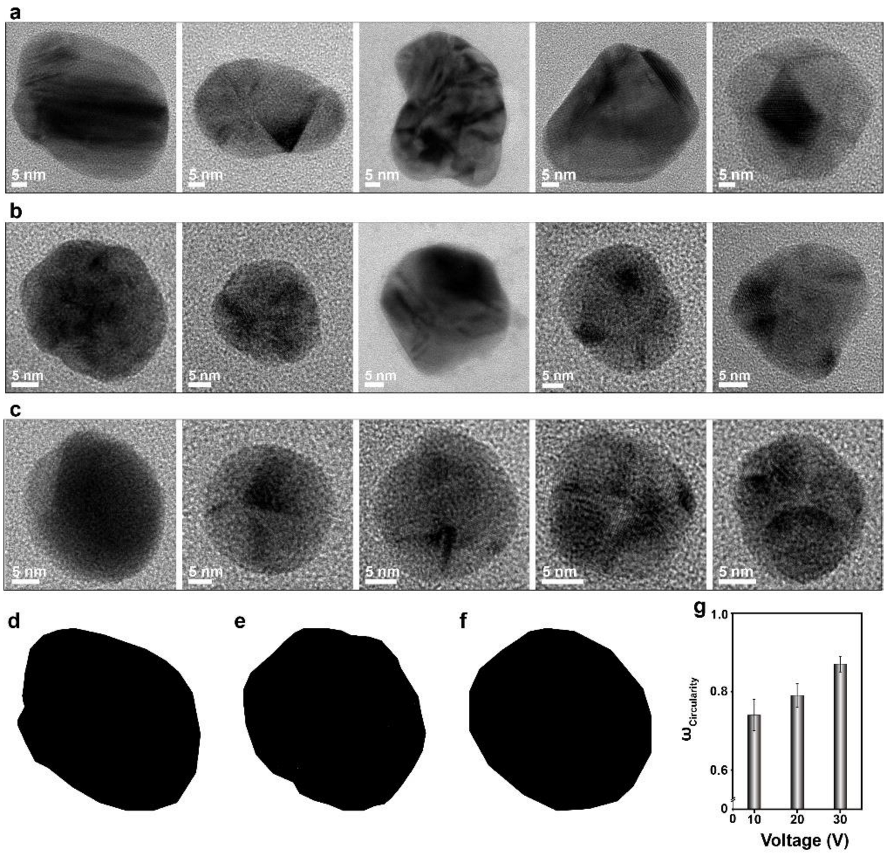

Supplementary Fig. 4. Surface topography of Ag nanoparticles prepared at different voltages. a to **c,** High resolution TEM images of the Ag nanoparticles dispersed in aqueous solutions prepared at 10 V, 20 V, and 30 V, respectively. **d** to **e**, The representative projections of the high resolution TEM images of Ag nanoparticles prepared at 10 V, 20 V, and 30 V, respectively. **g,** Quantitatively characterization of the surface roughness of the Ag nanoparticles prepared at different voltages. $\omega_{circularity} = 4\pi A / L$, where A is the projected area and L is the overall perimeter of the projected image of the Ag nanoparticles. $\omega_{circularity}$ is dependent on the shape of the projected image, being 1 for spherical spheres and less than 1 for any other shapes. Error bars represent standard deviation on the basis of five independent measurements.

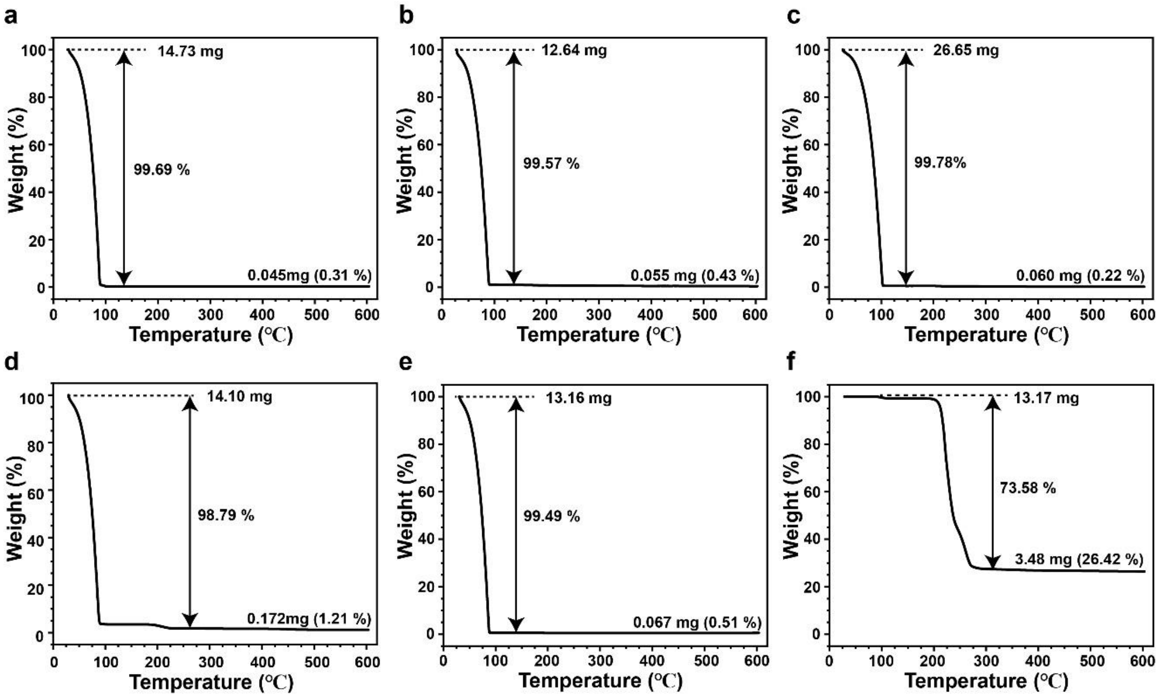

Supplementary Fig. 5. Thermogravimetric analysis (TGA) curves of Ag colloidal solutions prepared at different concentrations of SDS. a to **c**, TGA curves of electrochemically prepared Ag nanoparticles in the electrolyte solutions composed of 90 mM, 10 mM, and 3 mM SDS, respectively. The aluminum foil was used as the cathode. The initial mass of the colloidal solutions used for the TGA analysis was marked as the 100% weight. The left weight was that of the Ag nanoparticles and the decomposed product of SDS. **d**, TGA curves of electrochemically prepared Ag nanoparticles in the electrolyte solutions composed of 90 mM SDS using a carbon rod as the cathode. **e**, TGA curve of Ag nanoparticles prepared by the wet chemical method. **f**, TGA curve of the SDS powder.

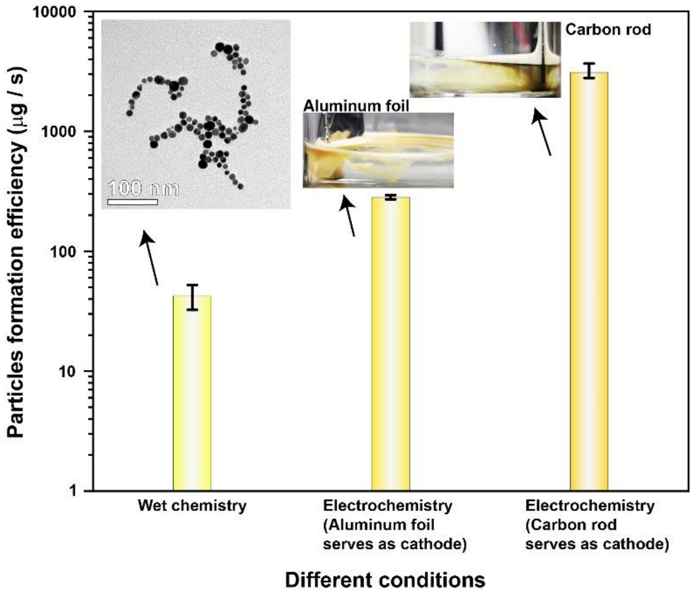

Supplementary Fig. 6. The yield efficiency of smart Ag nanoparticles using the wet chemical and the electrochemical method. Inset: TEM image of wet chemistry prepared Ag nanoparticles and photos of electrodeposition process of smart Ag nanoparticles using the aluminum foil and the carbon rod as the cathode electrode. Error bars represent standard deviation on the basis of five independent measurements.

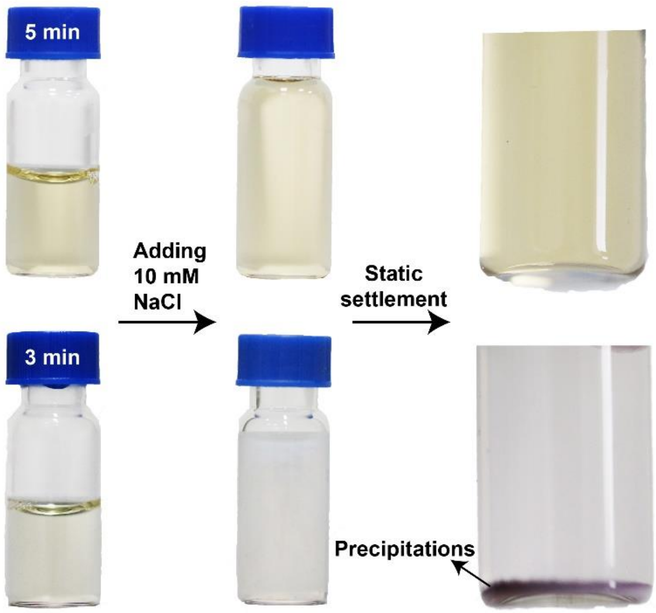

Supplementary Fig. 7. Consuming speed of silver ions during electrodeposition. After electrodeposition for 3 and 5 minutes, we removed the Ag nanoparticles by centrifugation. Introduction of 10 mM chloride ions to verify the existence of silver ions in the electrolyte.

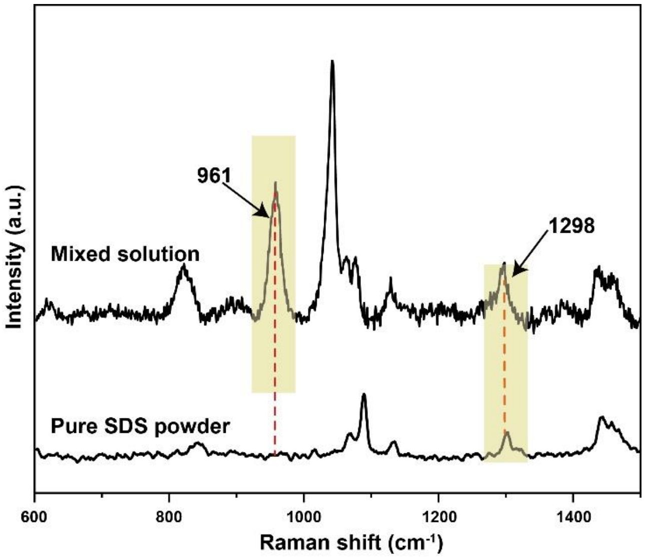

Supplementary Fig. 8. Raman characterization. Raman spectra of the aqueous solution composed of silver nitrate/SDS and pure SDS powder were measured. The appearance of the 961 cm^{-1} Raman peak indicates that there are strong interactions between silver ions and dodecyl sulfate ions.

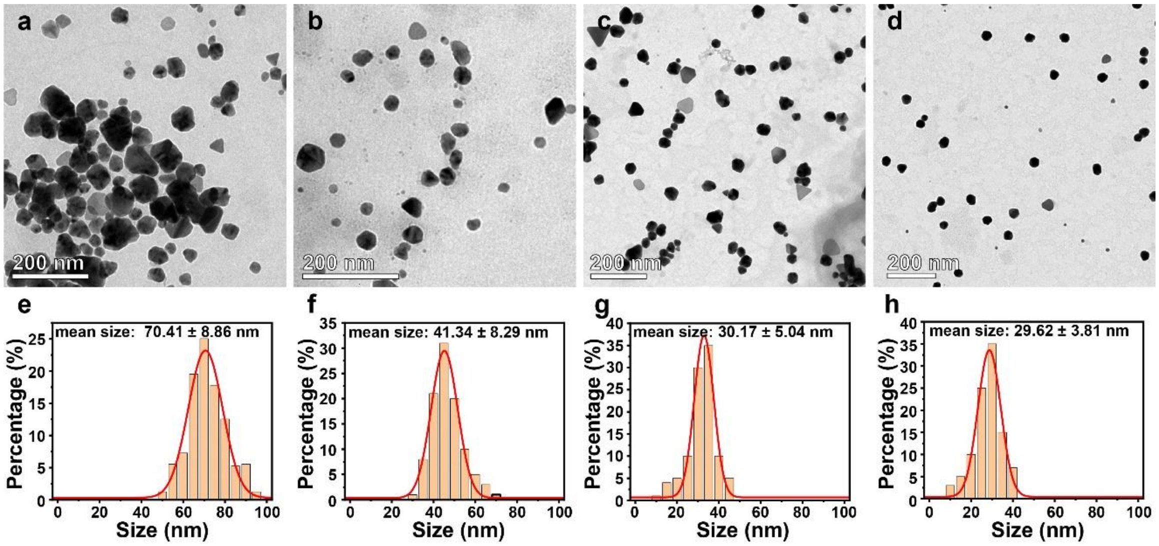

Supplementary Fig. 9. TEM images and size distribution of the smart Ag nanoparticles electrodeposited in the electrolyte with different molar ratios of SDS to silver nitrate. **a** to **d**, The molar ratio of SDS to silver nitrate is 0.5, 1.5, 3, and 4.5, respectively. **e** to **h**, Size distribution of the Ag nanoparticles based on the measurement of > 100 nanoparticles electrodeposited in the electrolyte with different molar ratios of SDS to silver nitrate at 0.5, 1.5, 3, and 4.5, respectively.

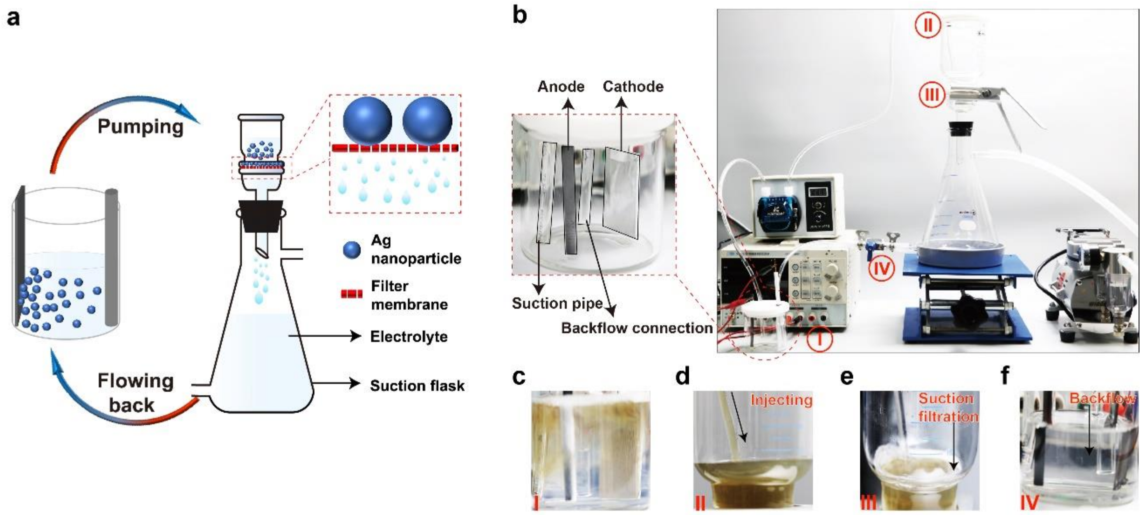

Supplementary Fig. 10. Automatic system constructed for continuous electrochemical preparation of the smart Ag nanoparticles. a, Schematic of the automatic fabrication system consists of four parts. **b**, The automatic fabrication system consists of four parts. **c**, Part I is the electrochemical cell used for preparation of Ag nanoparticles. **d**, Part II is the pumping apparatus used to transport the electrolyte composed of Ag nanoparticles into the filtration setup. **e**, The filtration setup. **f**, The transparent electrolyte in the filtration container is pumped back to the electrochemical cell.

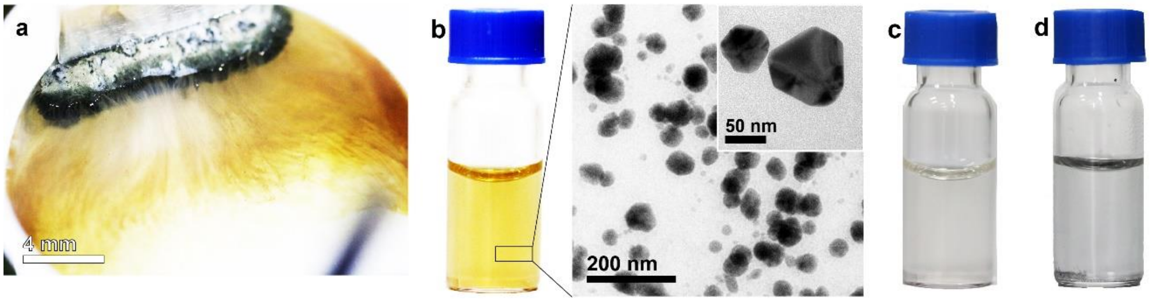

Supplementary Fig. 11. Electrochemical fabrication of Ag nanoparticles in the electrolyte composed of PVP. a, Yellow mist formed during electrodeposition at 30 V. **b**, Photo of the colloidal solution and the TEM image of the fabricated Ag nanoparticles. Inset: Magnified image. **c**, Ag nanoparticles precipitated at the bottom after 7 days. **d**, Aggregates immediately formed when freshly prepared Ag nanoparticles were transferred into toluene.

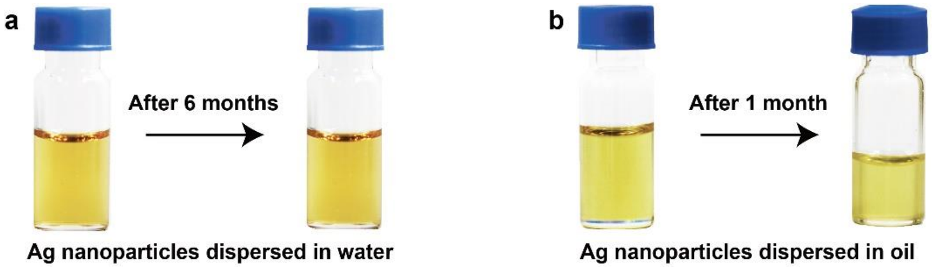

Supplementary Fig. 12. Colloidal stability of the smart Ag nanoparticles dispersed in water and toluene. a, **b**, No observable precipitates were observed within the colloidal solutions after 6 months in water and 1 month in toluene, indicating the good colloidal stability.

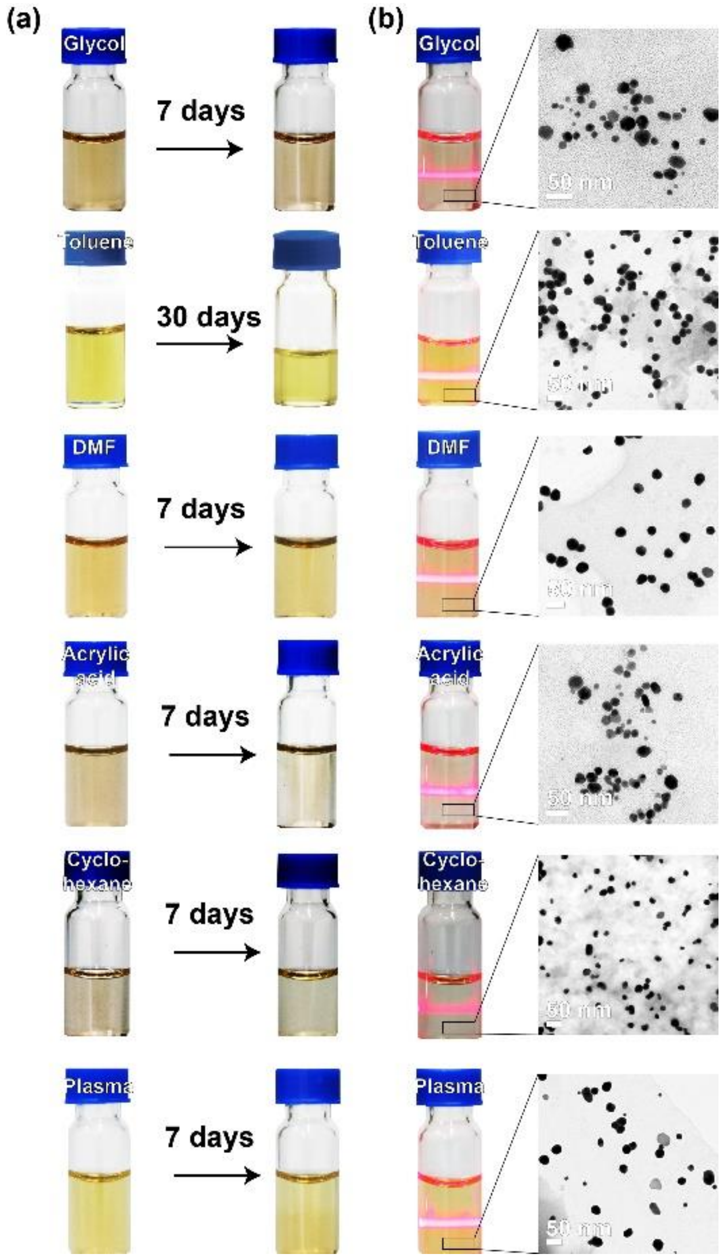

Supplementary Fig. 13. Long-term stability of the smart Ag nanoparticles dispersed in different liquids. a, Photos of silver colloidal solutions stored at room temperature for at least 7 days. **b,** Tyndall effect and TEM images of the Ag nanoparticles dispersed in different solvents for 7 days.

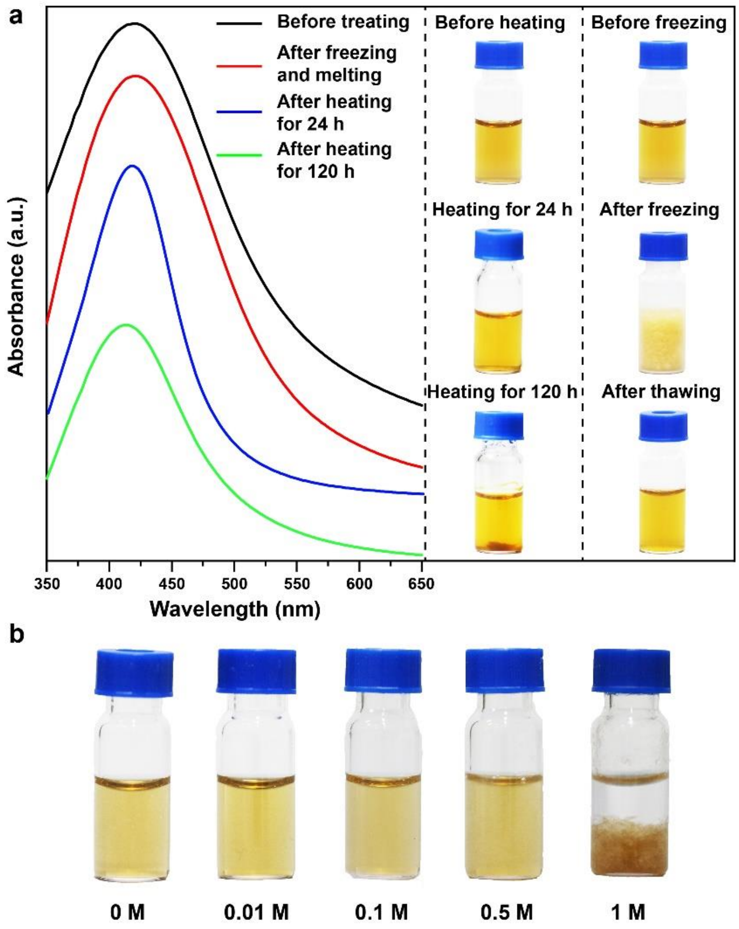

Supplementary Fig. 14. The stability of the aqueous colloidal solutions composed of smart Ag nanoparticles after different treatments. a, After heating at 90 ^{o}C for 24 h, no precipitates were observed. Only a small amount of aggregate was formed after heating for 120 h. After freezing and thawing, no precipitates were observed. **b,** Photos of the smart Ag nanoparticles dispersed in water composed of different amounts of sodium nitrate.

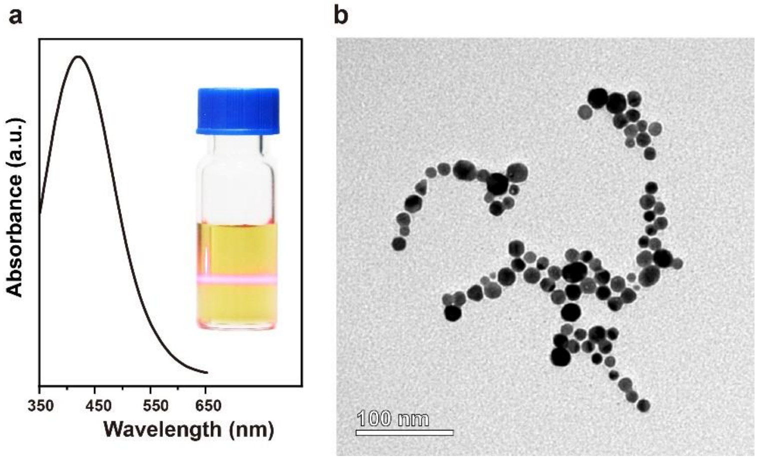

Supplementary Fig. 15. Ag nanoparticles prepared by wet chemical method. a, Absorption spectrum of the Ag nanoparticle colloid. Inset: Tyndall effect was observed when a laser beam passing through the colloidal solution. **b,** TEM image of the Ag nanoparticles prepared by wet chemical method.

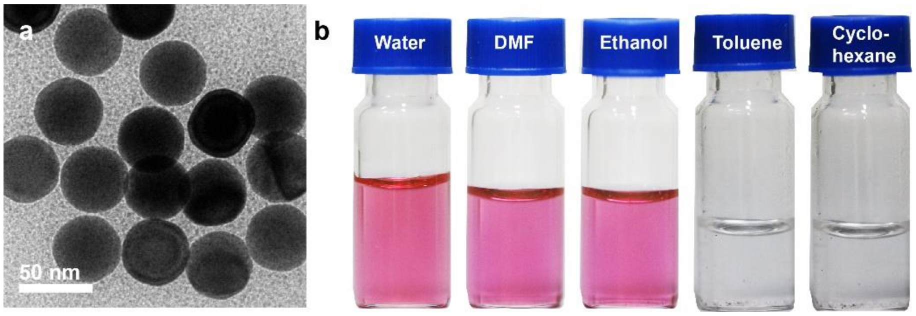

Supplementary Fig. 16. Dispersible limit of Au nanoparticles. a, TEM images of Au particles capped by hexadecyltrimethylammonium ligands. **b,** Au nanoparticles can disperse in water, DMF, and ethanol, while immediately aggregated in toluene and cyclohexane.

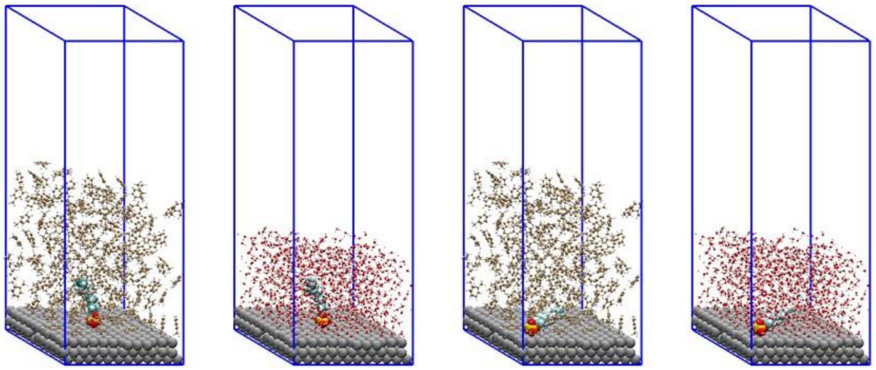

a b c d

Supplementary Fig. 17. Snapshots of the whole systems used in MD simulations. a, **b**, The ligand at standing state on Ag surface in toluene and water, respectively. **c**, **d**, The ligand at lying state on Ag surface in toluene and water, respectively.

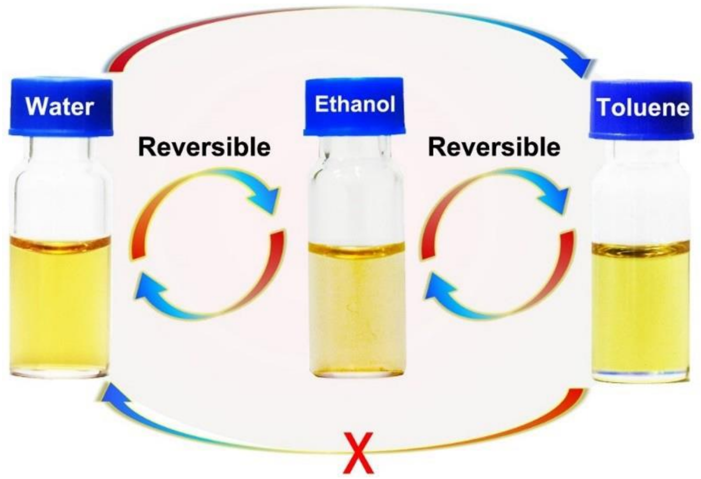

Supplementary Fig. 18. Anomalous dispersive properties of smart Ag nanoparticles. The smart Ag nanoparticles prepared in water could be centrifuged out and redispersed into ethanol, or vise versa. The smart Ag nanoparticles in ethanol could be centrifuged out and redispersed into toluene, or vise versa. The smart Ag nanoparticles prepared in water could be centrifuged out and redispersed into toluene; however, the nanoparticles in toluene after centrifuging out could not be redispersed into water.

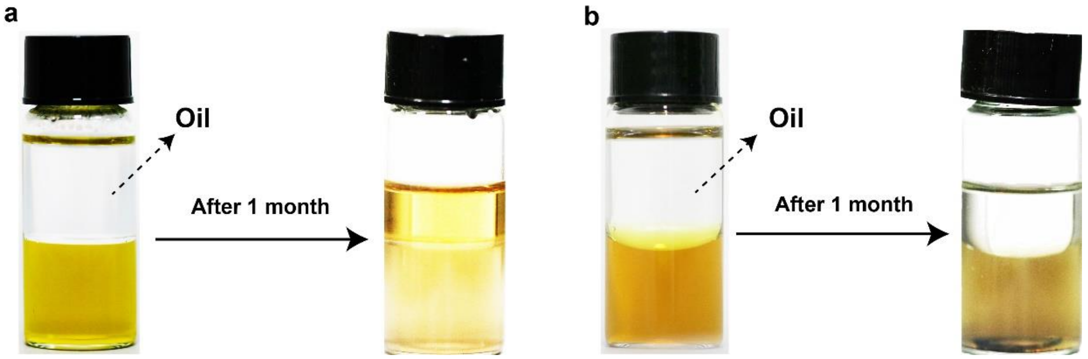

Supplementary Fig. 19. Immigration of smart Ag nanoparticles from water to oil (toluene). a, The smart Ag nanoparticles immigrated into toluene after 1 month. **b**, Wet chemistry prepared Ag nanoparticles precipitated at the bottom and failed to immigrate into toluene.

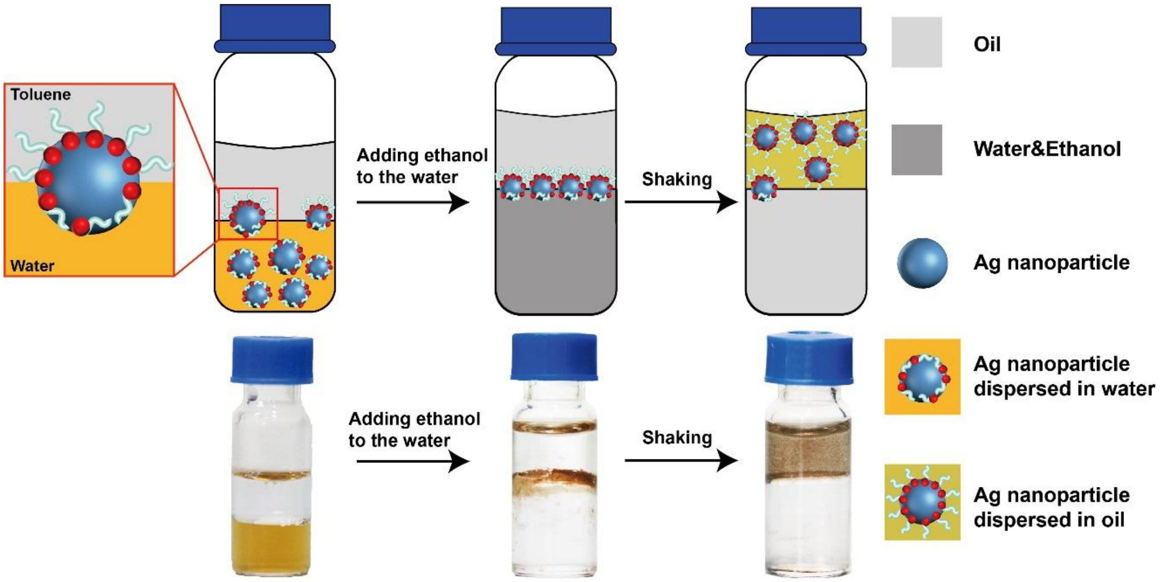

Supplementary Fig. 20. Trapping the smart Ag nanoparticles at the water/oil interface and transport them from water to oil phase (toluene). When ethanol was introduced into the colloidal solution, the Ag nanoparticles were immediately trapped at the water/toluene interface. Gently shaking the bottle helped the aggregated Ag nanoparticles to transport into toluene.

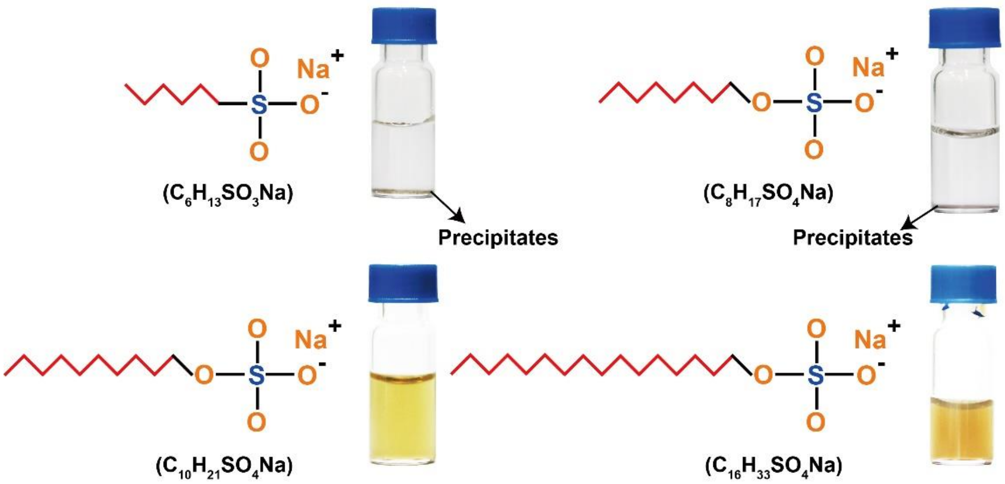

Supplementary Fig. 21. The critical alkyl chain length to allow the ligands to rotate. The alkyl chain should contain at least 10 carbon atoms to allow the ligands to rotate, enabling the Ag nanoparticles to disperse in both water and oil. Otherwise, the Ag nanoparticles cannot disperse in neither water nor oil.

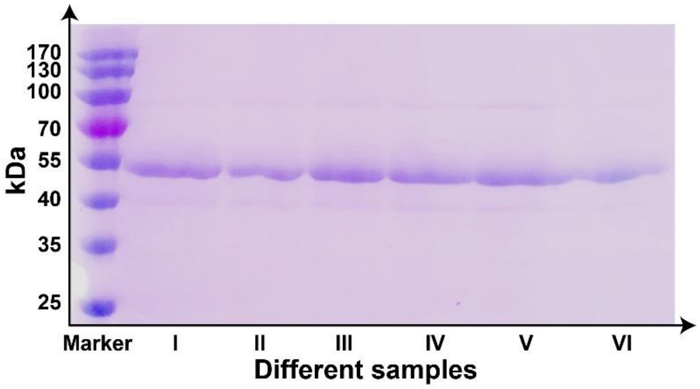

Supplementary Fig. 22. Characterization of flagellin. Coomassie brilliant blue stained SDS-Polyacrylamide gel electrophoresis of the flagellin prepared from *Salmonella* enterica serovar Typhimurium (ATCC14028) wild type.

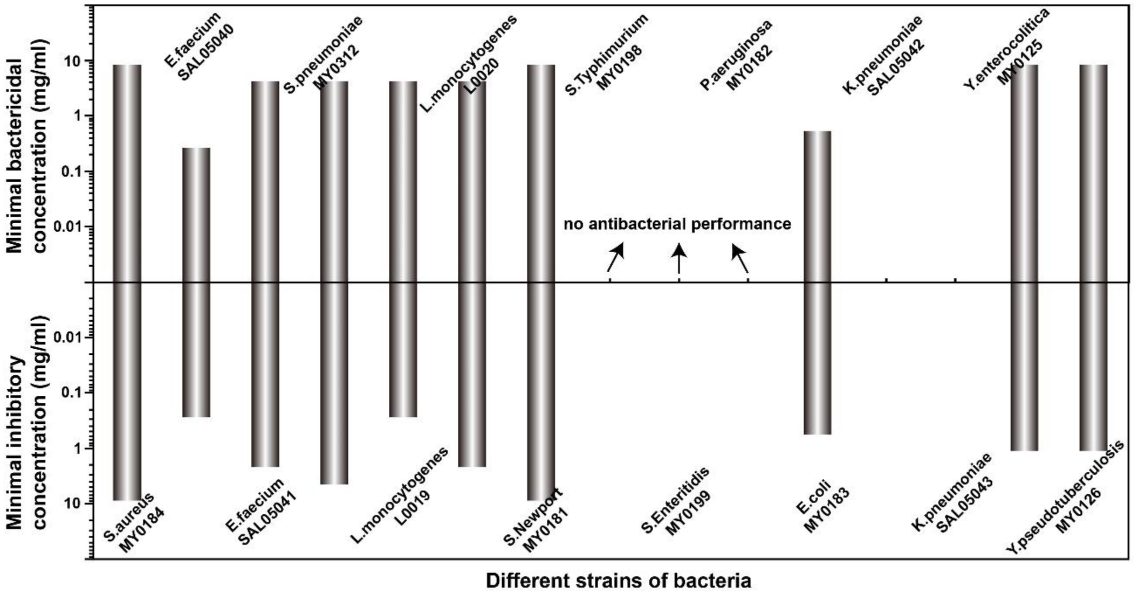

Supplementary Fig. 23. Evaluation of the antibacterial performance of pure SDS. The MIC and MBC of SDS toward different bacteria were shown. The dodecyl sulfate ligands without binding to the Ag nanoparticles have very weak antibacterial activity.

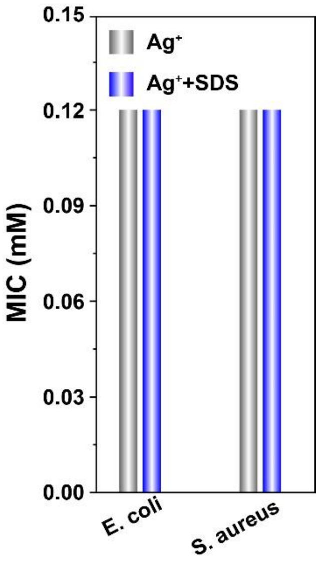

Supplementary Fig. 24. **Evaluation of the antimicrobial performance of Ag^+ ions and Ag^+ ions with SDS.** The MIC values towards *E. coli* and *S. aureus* were shown. SDS could not enhance the antibacterial performance of silver ions.

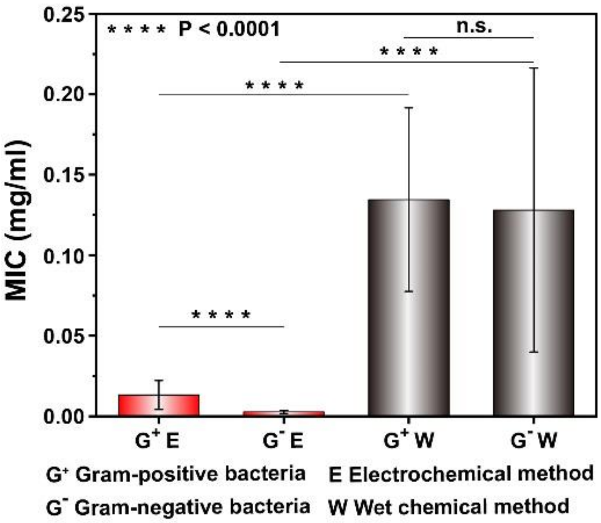

Supplementary Fig. 25. Antimicrobial performance of the smart Ag nanoparticles and the wet chemical method prepared ones toward gram-positive and gram-negative bacteria. Averaged MIC values of the smart Ag nanoparticles and the wet chemical method prepared ones toward gram-positive and gram-negative bacteria were shown. T-tests was used for significance calculating. n.s.: not significant. Error bars represent standard deviation on the basis of five independent measurements.

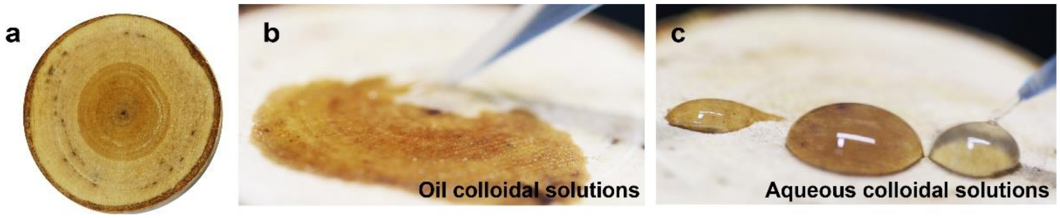

Supplementary Fig. 26. Dropping toluene and aqueous colloidal solutions on the wood surface. a, Photo of the wood before treatment. **b, c,** The toluene colloidal solution spread on the wood surface immediately, while it was difficult for the aqueous colloidal solution to spread on the wood.

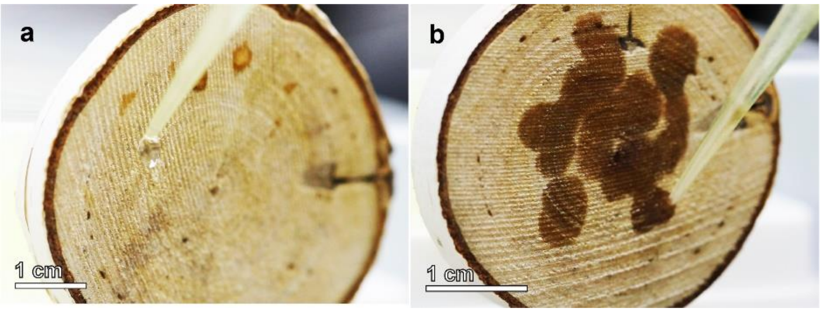

Supplementary Fig. 27. Dropping toluene and aqueous colloidal solutions on the vertically placed wood. a, b, Dropping the smart Ag nanoparticles dispersed in water and toluene onto the vertically placed wood. The aqueous colloidal solutions were hard to attach to the wood surface, while the toluene colloidal solutions easily attached to and got sucked by the wood.

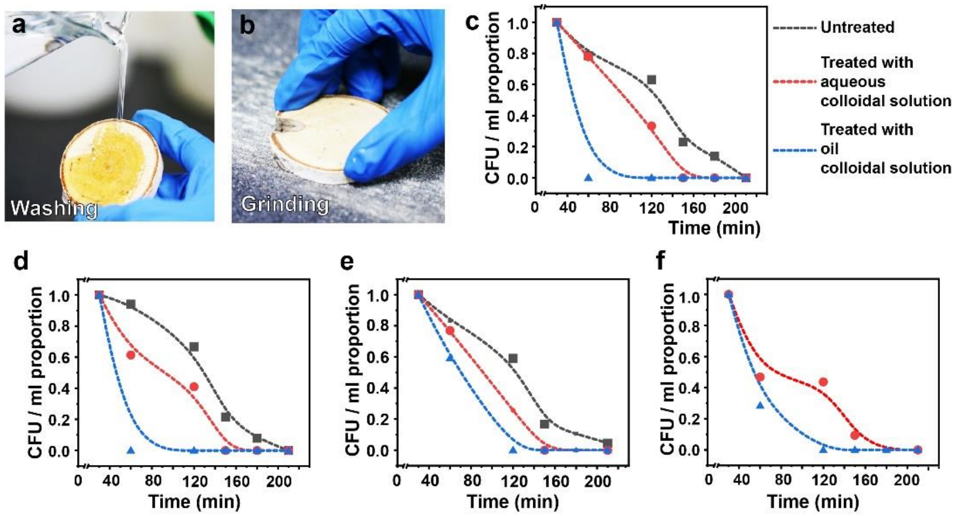

Supplementary Fig. 28. Stability of the smart Ag nanoparticle colloids as antimicrobial paints in air. a, b, Washing and grinding treatment of the smart Ag nanoparticles stained wood, respectively. **c,** Bacteria development on the pristine wood surface and after treatment with smart Ag nanoparticles dispersed in water and toluene. **d, e,** Bacteria development on the Ag nanoparticle-stained wood after water washing and grinding treatment, respectively. **f,** Bacteria development on the vertically placed wood surface after staining the smart Ag nanoparticles dispersed in water and oil.
