# Supplementary material for: Breaking the nanoparticle’s dispersible limit via rotatable surface ligands
Source: Nat Commun. 2022 Jun 23;13:3581. doi: 10.1038/s41467-022-31275-7 (PMC9226028; doi:10.1038/s41467-022-31275-7)
Supplement: Supplementary file 3 — Description of Additional Supplementary Files [file 41467_2022_31275_MOESM3_ESM.docx]

**Supplementary Movies**

Supplementary Movie 1 shows the continuous production of smart Ag nanoparticles in the electrolyte solution.

Supplementary Movie 2 shows no Ag nanoparticle formation in the electrolyte solution without SDS.

Supplementary Movie 3 shows the MD simulation result of the ligand rotation from standing to lying orientation when exposed to water.

Supplementary Movie 4 shows the MD simulation result of the ligand rotation from lying to standing orientation when exposed to toluene.

Supplementary Movie 5 shows pipetting the aqueous and oil colloidal solutions composed of smart Ag nanoparticles onto horizontally placed wood surface.

Supplementary Movie 6 shows spraying the aqueous and oil colloidal solutions composed of smart Ag nanoparticles onto vertically placed wood surface.
